# Characterization of the complete chloroplast genome sequence of *Malus toringoides* (Rosaceae)

**DOI:** 10.1080/23802359.2020.1781572

**Published:** 2020-06-26

**Authors:** Shuai Wang, Xiaoran Cai, Bin Zhang, Hongwei Wang, Fude Shang

**Affiliations:** a College of Life Science, Henan Agricultural University, Zhengzhou, China; b College of Plant Protection, Henan Agricultural University, Zhengzhou, China

**Keywords:** *Malus toringoides*, chloroplast genome, Rosaceae, phylogenetic analysis

## Abstract

*Malus toringoides* belongs to the *Malus* genus (Rosaceae) and is a precious resource among wild plants. In this study, we report the first complete chloroplast (cp) genome sequence of *M. toringoides*. The whole cp genome contains 126 genes, 83 protein-coding genes, 35 tRNA genes, and eight rRNA genes. A maximum-likelihood phylogenetic tree analysis based on 12 complete chloroplast genomes indicated that *M. toringoides* clustered closely with *Malus hupehensis*. Thus, the chloroplast genome can provide valuable genetic information for the protection and exploitation of *M. toringoides*.

*Malus toringoides* (Rehd.) Hughes, belonging to the *Malus* genus (Rosaceae), is endemic to the Hengduan Mountains, China (Shi et al. 2004). This species has a well-developed root system and is highly resistant to stress, so it is a good tree species for ecological restoration of degraded vegetation in arid alpine areas (Xu et al. 2011). *Malus toringoides* is also an excellent apple rootstock with good grafting compatibility, resulting in strong growth of grafted seedlings, a semidwarfing habit, early fruiting, high yields and good fruit quality (Shi et al. 2004). In addition, this species has other attributes, such as high nutritional value of the fruit, the ability to make its leaves into tea, and significant anti-inflammatory activity of the total flavonoids extracted from its leaves (Wang et al. 2009; Chang et al. 2019). In this study, we report the complete chloroplast (cp) genome to provide valuable genetic information for the protection and exploitation of *M. toringoides*.

Fresh leaf samples were collected from plants at the Beijing Institute of Botany (39°59′25″N, 116°12′34″E, alt. 72 m), Beijing city, China. The specimens (WS20190728) have been preserved in the herbarium of Henan Agricultural University. Total genomic DNA was extracted from the leaves of *M. toringoides* using the modified cetyl trimethyl ammonium bromide (CTAB) method (Fang et al. 2009). The purified DNA was then fragmented and used to construct short-insert libraries (insert size of 430 bp) according to the manufacturer’s instructions (Illumina) and then sequenced on an Illumina HiSeq 4000 (Borgstrom et al. 2011). After the raw reads were filtered, the clean reads were assembled into circular genomes by GetOrganelle (Jin et al. 2018) using the cp genome of *Malus sieversii* (MK434920) as a reference. Annotations and adjustments of the genes were conducted manually using Geneious Prime (Kearse et al. 2012). The validated complete cp genome sequence was ultimately submitted to GenBank (accession number: MT442040).

The complete cp genome of *M. toringoides* is 160,094 bp in length and contains a large single-copy (LSC) region of 88,184 bp, a small single-copy (SSC) region of 19,194 bp, and two inverted repeat (IR) regions of 26,358 bp. The whole sequence of *M. toringoides* contains 126 genes, including 83 protein-coding genes, 35 tRNA genes, and eight rRNA genes. The overall GC content of the whole plastid genome is 36.55%, with the corresponding values of the LSC, SSC, and IR regions being 34.22%, 30.39%, and 42.70%, respectively.

To analyze the phylogeny, the complete chloroplast genome sequences from 12 species were aligned via the MAFFT method of Geneious Prime (Kearse et al. 2012). Based on all the above data, we constructed a maximum-likelihood (ML) tree using *Chaenomeles sinensis* as an outgroup. The ML phylogenetic results indicated that *M. toringoides* is closely related to *M. hupehensis*, with high bootstrap support (Figure 1). The whole chloroplast genome of *M. toringoides* provides useful genetic information for molecular marker development and phylogenetic studies, and these results can be further used for the conservation and exploitation of this species.

**Figure 1.**
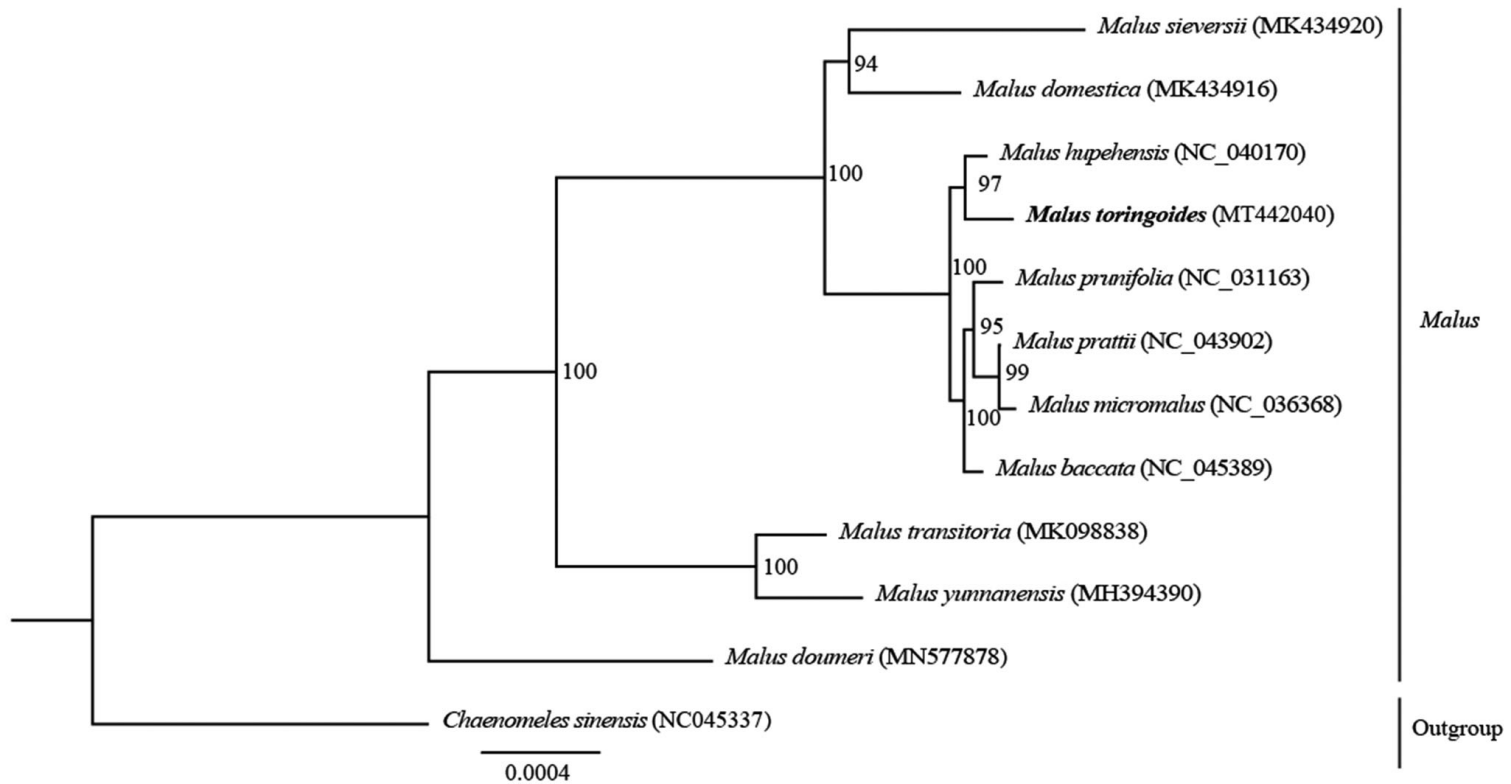
ML phylogenetic tree construction comprising 12 species based on complete chloroplast genome sequences. Bootstrap support values are shown beside the nodes. The new complete cp genomes obtained in this study are shown in bold.

## Data Availability

The data supporting the findings of this study are publicly available in the National Center for Biotechnology Information (NCBI) database at https://www.ncbi.nlm.nih.gov/ under reference number MT268884.
